# Supplementary material for: A homing suppression gene drive with multiplexed gRNAs maintains high drive conversion efficiency and avoids functional resistance alleles
Source: G3 (Bethesda). 2022 Apr 8;12(6):jkac081. doi: 10.1093/g3journal/jkac081 (PMC9157102; doi:10.1093/g3journal/jkac081)
Supplement: jkac081_Supplementary_Data [file jkac081_supplementary_data.zip › Supplemental_Material_G3-2022-403336.pdf]

A homing suppression gene drive with multiplexed gRNAs maintains high drive conversion efficiency and avoids functional resistance alleles

Emily Yang[1,2], Matthew Metzloff[1,2], Anna M. Langmüller[1,3,4], Xuejiao Xu[5], Andrew G. Clark[1,2], Philipp W. Messer[1], Jackson Champer[1,2,5*]

[1]Department of Computational Biology, Cornell University, Ithaca, NY 14853
[2]Department of Molecular Biology and Genetics, Cornell University, Ithaca, NY 14853
[3]Institut für Populationsgenetik, Vetmeduni Vienna, Veterinärplatz 1, 1210 Wien, Austria
[4]Vienna Graduate School of Population Genetics, 1210 Wien, Austria
[5]Current Address: Center for Bioinformatics, School of Life Sciences, Peking-Tsinghua Center for Life Sciences, Peking University, Beijing, China 100871

*Corresponding author: JC (jchamper@pku.edu.cn)

SUPPLEMENTARY INFORMATION

Supplementary Methods

Plasmid Construction:

gRNA-tRNA Array:

**TTTygU4**	*Template*	*Oligo/Enzyme 1*	*Oligo/Enzyme 2*
PCR Product	TTTgRNAtRNAi	YGg_g41_F	YGg_g41_R
PCR Product	TTTgRNAt	YGg_g12_F	YGg_g12_R
PCR Product	TTTgRNAt	YGg_g23_F	YGg_g23_R
PCR Product	TTTgRNAt	YGg_g34_F	YGg_g34_R

Left Homology Arm:

**HSDygU4i1**	*Template*	*Oligo/Enzyme 1*	*Oligo/Enzyme 2*	*Enzyme 3*
PCR Product	Genomic DNA	YGLeft_F	YGLefth_R	N/A
Plasmid Digest	BHDrN1*	KpnI	NotI	NheI

*Similar to previously constructed plasmid BHDcN1[20] for purposes of cloning in this study, but with the following sequence immediately 5’ of the ApaI site sequence:

```
CACACTGTGTGTGCAGCTCGAGGCTCTTCCGTCAATCAAGTTCAAGGGCGACACAAAATTTATTCTAAATGCATAAT
AAATACTGATAACATCTTATAGTTTGTATTATATTTTGTATTATCGTTGACATGTATAATTTTGATATCAAAAACTG
ATTTTCCCTTTATTATTTTCGAGATTTATTTTCTTAATTCTCTTTAACAAACTAGAAATATTGTATATACAAAAAAT
CATAAATAATAGATGAATAGTTTAATTATAGGTGTTCATCAATCGAAAAAGCAACGTATCTTATTTAAAGTGCGTTG
CTTTTTTCTCATTTATAAGGTTAAATAATTCTCATATATCAAGCAAAGTGACAGGCGCCCTTAAATATTCTGACAAA
TGCTCTTTCCCTAAACTCCCCCCATAAAAAAACCCGCCGAAGCGGGTTTTTACGTTATTTGCGGATTAACGATTACT
CGTTATCAGAACCGCCCAGG
```

Right Homology Arm and gRNA:

**HSDygU4**	*Template*	*Oligo/Enzyme 1*	*Oligo/Enzyme 2*
PCR Product	Genomic DNA	YGRight_F	YGRighth_R
PCR Product	TTTygU4	gRNA_SV40_F	gRNA_YG_R
Plasmid Digest	HSDygU4i1	MluI	XbaI

Construction primers

HSDygU4i1:

YGLeft_F: TAGGGGTCAGTGTTACAACCAATTAACCAGGTACCGTGGGTGGATTACAGGGTAGCA
YGLefth_R: TTAGTCTCTAATTGAATTAGATCCGCGGCCGCCTGCGGATGGGCTGCTCC

HSDygU4:

YGRight_F: TTTAATGTTCGCTTAATGCGTATGCATAGGCCTCCAAGGACAACAAGCCATTCG
YGRighth_R: GGCATCAAACTAAGCAGAAGGCCCCTGACTCTAGAGTGGAGGGATACGGACTCAA
gRNA_SV40_F: GGTTTGTCCAAACTCATCAATGTATCTTAACGCGTTTTTTTGCTCACCTGTGATTGCTC
gRNA_YG_R: CCTATGCATACGCATTAAGCGAACA

TTTygU4:

YGg_g41_F: GTGCACATAAACACGGCCAACCACAGTTTTAGAGCTAGAAATAGCAAGTTAAA
YGg_g41_R: AAAACCAGATGCAGTCCCAAGATCGTGCATCGGCCGGGAATCG
YGg_g12_F: GCACGATCTTGGGACTGCATCTGGTTTTAGAGCTAGAAATAGCAAGTTAAA
YGg_g12_R: AACTGGAGTAGTCGACCACGATGTGCACCAGCCGGGAATCG
YGg_g23_F: GCACATCGTGGTCGACTACTCCAGTTTTAGAGCTAGAAATAGCAAGTTAAA
YGg_g23_R: AACGGTCACCTCCGAGAGTCGGCTGCACCAGCCGGGAATCG
YGg_g34_F: GCAGCCGACTCTCGGAGGTGACCGTTTTAGAGCTAGAAATAGCAAGTTAAA
YGg_g34_R: TGTGGTTGGCCGTGTTTATGTGCACCAGCCGGGAATCG

Sequencing primers (for confirming plasmid sequences and sequencing resistance alleles)

EGFPaLeft_S_R: GCGAAAGCTAAGCAAATAAACAAGC
U6term_S_F: CATCTGACGTGTGTTTATTTAGAC
Yellow_gRNA1_S_F: TTGCTCACCTGTGATTGCTCC
CFD5_S_R: TAGACAATGGTTTTCCGTTGACGT
YGLeft_S_F: ACAAACGGCAAACAAACGAGG
YGLeft_S_R: TGGCGGCTAATTGAAATGTTGG
YGRight_S_F: TCGAACTGAATCAAGAGTTTGGAG
YGRight_S_R: TGAGCCACACTTCTGAGAACT

Supplementary Results

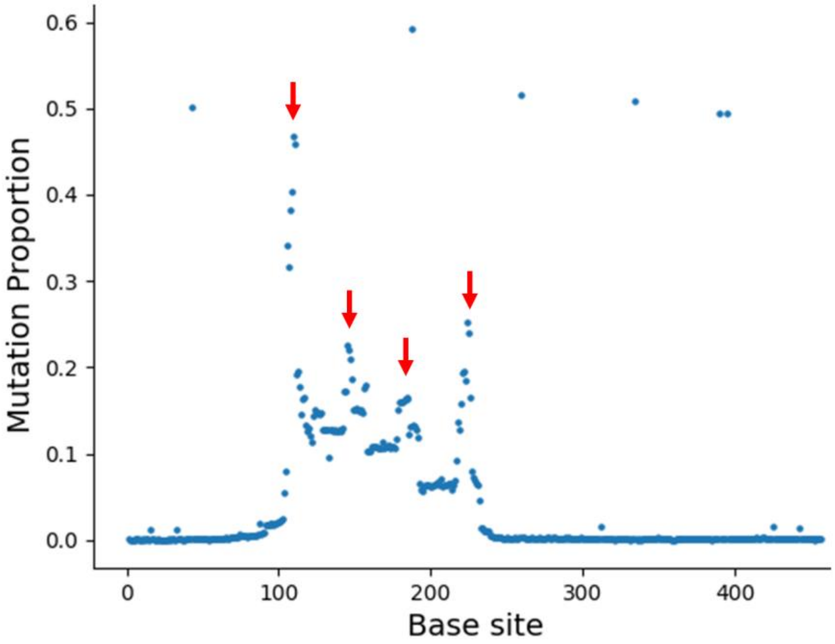

Figure S1. Mutations at gRNA cut sites. Several female and male drive heterozygotes were crossed to each other, and approximately 100 progeny were collected. Pooled DNA was purified and used as a template for PCR around the drive target site. PCR products were analyzed by deep sequencing. The chart shows the fraction of DNA at each nucleotide that was different from the wild-type allele. Arrows show locations of the four gRNA target sites.

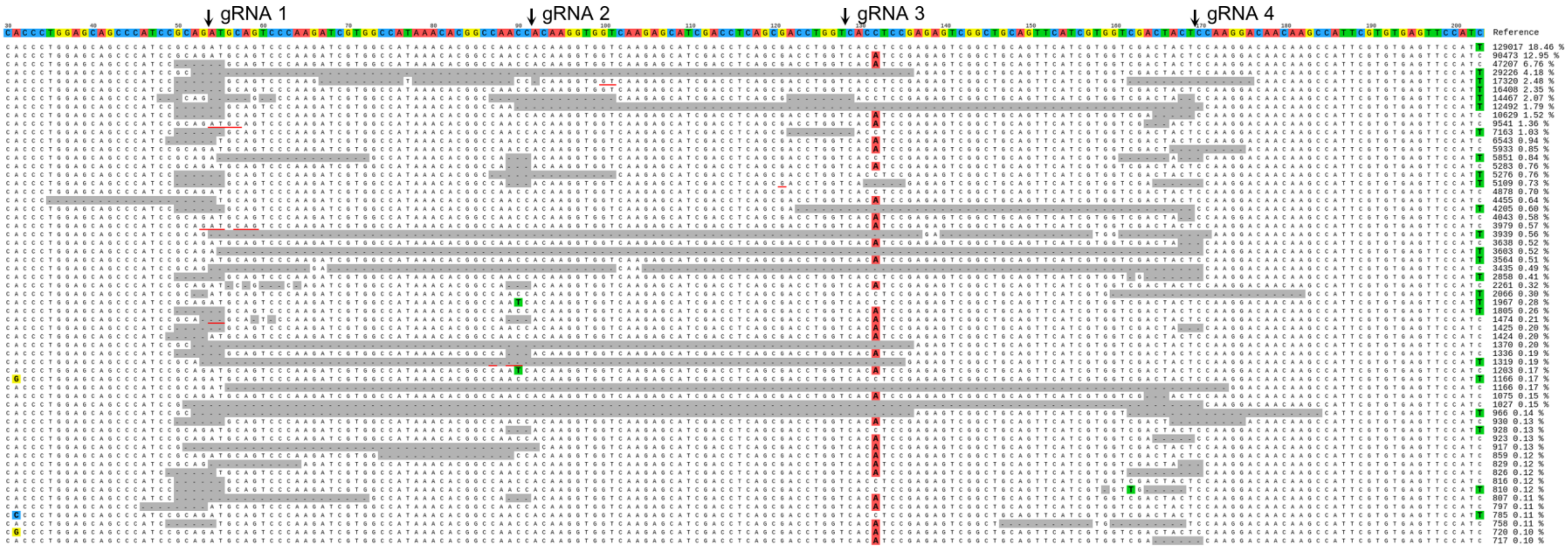

Figure S2. Resistance allele sequences. Several female and male drive heterozygotes were crossed to each other, and approximately 100 progeny were collected. Pooled DNA was purified and used as a template for PCR around the drive target site. PCR products were analyzed by deep sequencing. Arrows above each panel show the gRNA cut sites. Highlighted nucleotides are different from the reference sequence. The "A" variant near gRNA target site 3 may be an error since it was not detected in any Sanger sequencing sample, but it could also represent a pre-existing resistance allele for gRNA #3 present in the population at intermediate frequency. Note that sequences with one or more small inserts interrupting large deletions were likely indels over the entire spanned region with a small insertion that formed during end-joining repair. Thus, the highlighted "T" nucleotides at gRNAs 2 and 3 simply represent sequences of an insertion after end-joining repair.

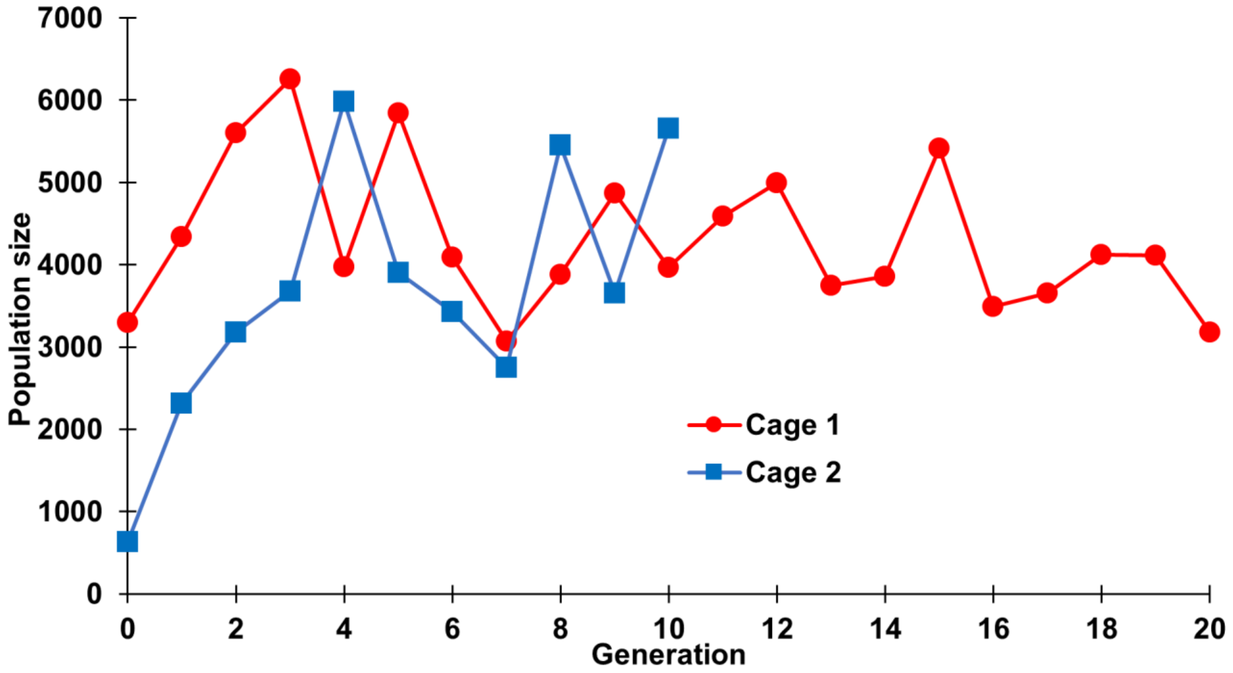

Figure S3. Cage population sizes. The population size for each generation is displayed for the two drive experiment cages from Figure 4.

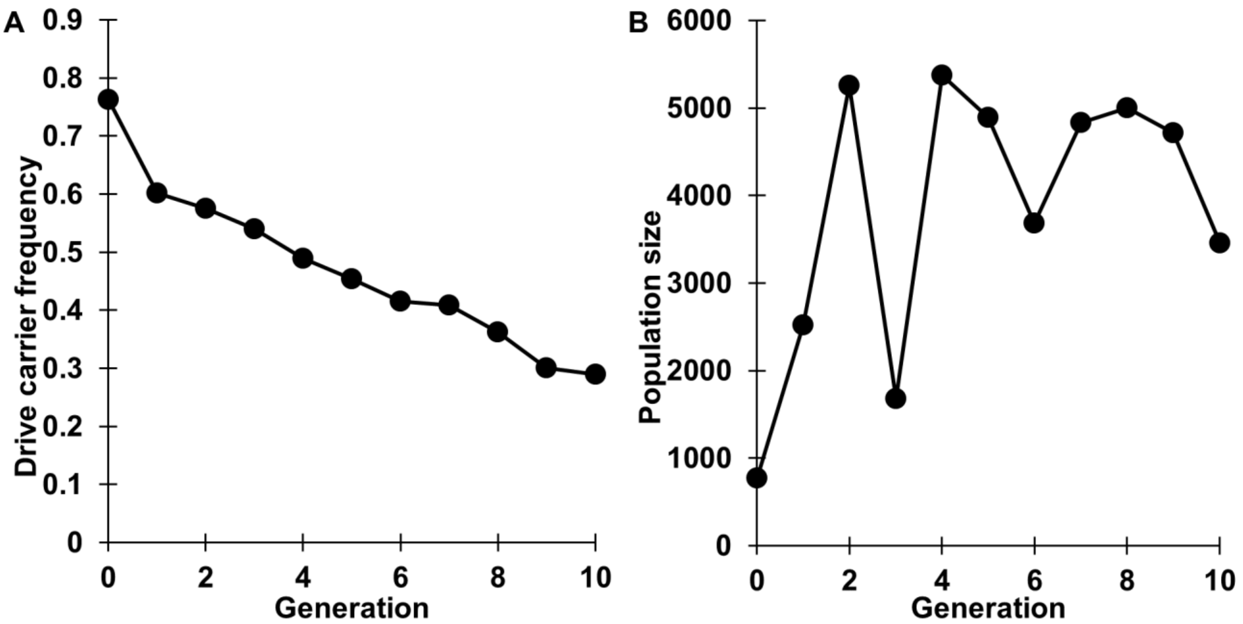

Figure S4. Cage experiment without Cas9. (**A**) Individuals with a drive allele and without Cas9 were introduced into a w^{1118} cage population without Cas9 at a carrier frequency of 76%. The cage population was followed for several non-overlapping generations, each lasting twelve days, including one day of egg-laying. All individuals from each generation were phenotyped for DsRed, with positive drive carriers having either one or two drive alleles. The drive allele cannot perform drive conversion, so it decreases in the population over time. (**B**) Size of the cage population in each generation.

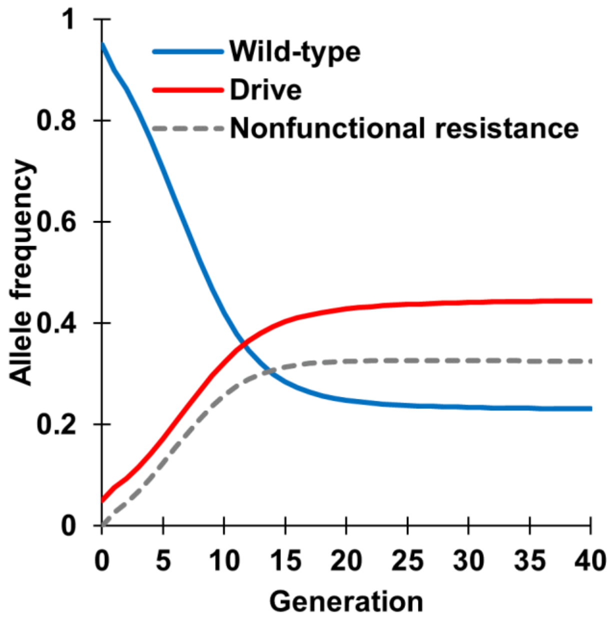

Figure S5. Predicted allele frequencies. Using the same drive efficiency parameters as modeled in our maximum likelihood study and an example female somatic fitness of 0.67, we plotted drive, wild-type, and nonfunctional resistance allele frequency trajectories. Equilibrium is nearly reached after 20 generations.

Table S1. Target site conservation

DNA sequence corresponding to target region (all NCBI reference sequences).

>*Drosophila melanogaster*
ATCCGCAGATGCAGTCCCAAGATCGTGGCCATAAACACGGCCAACCACAAGGTGGTCAAGAGCATCGACCTCAGCGACCTGGTCACCTCCGAGAGTCGGCTGCAGTTCATCGTGGTCGACTACTCCAAGGAC
>*Drosophila simulans*
ATCCGCAGATGCAGTCCCAAGATCGTGGCCATAAACACGGCCAACCACAAGGTGGTCAAGAGCATCGACCTCAGCGACCTGGTCACCTCCGAGAGTCGCCTGCAGTTCATCGTAGTCGACTACTCCAAGGAC
>*Drosophila suzukii*
ATCCGGAGGTGCAGCCCCAAGATCGTGGCCATAAACACCTCCAACAACAAGGTGGTCAAGAGCATCGACCTGAGTGACCTGGTCACCTCCGAGAGCCGCCTGCAGTTCATCGTGGTCGACTACTCCAAGGAC
>*Drosophila pseudoobscura*
ATCCGTCGCTGTGGCCCCAAAATAGTGGCCATCAACACGGCCAACCACAAGGTGGTGAAGAGCATCGATCTGAGCGATCTGGTGACCGCCGAGAGTCGACTGCAGTTCATAGTCGTCGACTATTCCAAGGAC
>*Drosophila virilis*
ATACGCCGTTGCAGCCCCAAAATTGTGGCCATTAATACGGCCAACAACAAGGTGGTGAAAAGCATCGATCTTAGCGATTTGGTGACCTCCGAAAGTCGTTTGCAGTTCATTGTCGTTGATTACTCCAAGGAT

gRNA target sites including PAM are underlined in *D. melanogaster*. Highlighted nucleotides are different from *D. melanogaster*.

Amino acid sequence corresponding to target region.

>*Drosophila melanogaster*
IRRCSPKIVAINTANHKVVKSIDLSDLVTSESRLQFIVVDYSKD
>*Drosophila simulans*
IRRCSPKIVAINTANHKVVKSIDLSDLVTSESRLQFIVVDYSKD
>*Drosophila suzukii*
IRRCSPKIVAINTSNNKVVKSIDLSDLVTSESRLQFIVVDYSKD
>*Drosophila pseudoobscura*
IRRCGPKIVAINTANHKVVKSIDLSDLVTAESRLQFIVVDYSKD
>*Drosophila virilis*
IRRCSPKIVAINTANNKVVKSIDLSDLVTSESRLQFIVVDYSKD

Amino acids with codons in or adjacent to the cut site are underlined in *D. melanogaster*. Highlighted amino acids are different from *D. melanogaster*.

(The corresponding proteins in mosquitoes have ~40-60% amino acid sequence identity and very little nucleotide sequence identity compared to *D. melanogaster*)

Table S2 Predicted off-target sites

Off-target sites were predicted with Brown University CRISPR Optimal Target Finder as noted in the drive construct design section. No strong off-target sites were predicted for each gRNA, but several sites were predicted using maximum stringency (gRNAs below are listed in order of their cut site in *yellow-g*).

gRNA1 (CGATCTTGGGACTGCATCTG*CGG*)
lncRNA:CR44066 possible promoter region
CG43980 coding exon
lncRNA:bxd intron (~200 nt from exons)
Mur89 intron (far from exons)
CG4362 intron/exon boundary
nmo intron (far from exons)
lncRNA:CR44066 possible promoter region

gRNA2 (CATAAACACGGCCAACCACA*AGG*)
CG42502 coding exon in some variants
Pgant9 coding exon
Intergenic region

gRNA3 (GCCGACTCTCGGAGGTGACC*AGG*)
Dscam1 coding exon in one variant (intron in most)
CG8925 coding exon

gRNA4 (CATCGTGGTCGACTACTCCA*AGG*)
AGO3 intron (far from exons, two separate sites)
CG13562 coding exon
CG4563 coding exon

Table S3. Resistance allele analysis

Progeny of drive heterozygote females or males were sequenced around the target site to identify resistance alleles. For sequence type, "E" indicates that the sequenced progeny is a drive carrier with a drive heterozygote mother. The sequence is therefore an embryo resistance allele. "Fg" indicates that the sequenced individual did not inherit a drive allele from a drive heterozygous mother. These could have two resistance alleles, one from germline and embryo cutting and the other from embryo cutting. "Mg" is similar, but has a male parent, so only germline cutting could take place. For "Fg" and "Mg", two alleles would be sequenced, and wild-type alleles are not displayed at each cut site unless they are the only one present. WT = wild-type. R = resistance. "-" indicates deletion between the sites. "*" indicates large deletion that continues past the target site. m = mosaic sequence. The high molecular weight product has the sequence displayed above, while the low molecular weight product for both consisted of a large deletion that went well beyond both outer gRNA target sites.

Sequence type	Cut site 1	Cut site 2	Cut site 3	Cut site 4	# of sequences with pattern
E	WT	WT	WT	WT	6
E	mosaic[1]	WT	WT	WT	5
E	mosaic	WT	WT	mosaic	2
E	WT	WT	WT	mosaic	1
E	mosaic	WT	mosaic	WT	1
E	R[1]	WT	WT	WT	4
E	WT	WT	WT	R	2
E	R-	-	-	*	1
E	R	R	WT	WT	1
E	mosaic	mosaic	mosaic	R	1
E	mosaic	WT	R	WT	1
Fg	R	WT	R	R	1
Fg	*-	R	WT	R	1
Fg	mosaic	WT	WT	WT	1
Mg	WT	WT	WT	WT	4
Mg	R	WT	WT	WT	2
Mg	R	R	-	R	1

Table S4. Maximum likelihood parameter estimates from cage populations

Fitness values are for drive homozygotes (multiplicative fitness per allele), except for somatic Cas9 cleavage, where the value is applied directly to drive/wild-type females. 1 is equivalent to wild-type. [Brackets] show 95% confidence intervals. All models have an effective population size parameter, and models with fitness costs have a single additional fitness parameter.
Log-likelihood: shows a relative probability (higher values indicate a better model fit)
AICc: Akaike information criterion, corrected (low values indicate a better match of the model without overfitting).

Table S4A. Cage 1 drive released into Cas9 background

**Fitness cost model**	**Log-likelihood**	**AICc**	**Effective population size**	**Fitness**
**None**	32.0	-61.7	115 [57-205]	1
**Somatic cleavage female fecundity**	38.1	-71.4	219 [108-388]	0.67 [0.54-0.81]
**Direct fecundity and mating**	38.7	-72.7	233 [115-415]	0.78 [0.70-0.88]
**Direct viability**	39.0	*-73.3*	241 [118-428]	0.80 [0.72-0.88]
**Off-target fecundity and mating**	35.6	-66.5	169 [83-300]	0.69 [0.57-0.86]
**Off-target viability**	35.6	-66.4	168 [83-298]	0.68 [0.56-0.86]

Table S4B. Cage 2 drive released into Cas9 background

**Fitness cost model**	**Log-likelihood**	**AICc**	**Effective population size**	**Fitness**
**None**	10.5	-18.4	44 [15-97]	1
**Somatic cleavage female fecundity**	18.0	*-30.1*	229 [77-509]	0.43 [0.30-0.57]
**Direct fecundity and mating**	15.9	-25.9	143 [48-319]	0.63 [0.51-0.77]
**Direct viability**	15.8	-25.6	140 [47-311]	0.66 [0.55-0.79]
**Off-target fecundity and mating**	16.6	-27.2	166 [56-370]	0.53 [0.42-0.67]
**Off-target viability**	17.1	-28.3	188 [63-418]	0.51 [0.42-0.64]

Table S4C. Cages 1 and 2 combined analysis

Fitness cost model	Log-likelihood	AICc	Effective population size	Fitness
**None**	40.9	-79.6	75 [43-122]	1
**Somatic cleavage female fecundity**	53.5	*-102.5*	184 [104-298]	0.57 [0.47-0.69]
**Direct fecundity and mating**	52.3	-100.2	169 [95-274]	0.73 [0.65-0.81]
**Direct viability**	52.5	-100.6	172 [97-278]	0.75 [0.68-0.82]
**Off-target fecundity and mating**	50.8	-97.2	152 [86-246]	0.62 [0.53-0.72]
**Off-target viability**	51.0	-97.6	154 [87-250]	0.61 [0.53-0.72]

Table S4D. Drive without any Cas9 present

Fitness cost model	Log-likelihood	AICc	Effective population size	Fitness
**None**	22.8	*-43.0*	635 [214-1414]	1
**Somatic cleavage female fecundity**	22.8	-39.5	635 [214-1414]	1.00 [0.90-1.22]
**Direct fecundity and mating**	22.8	-39.6	635 [214-1414]	1.00 [0.93-1.17]
**Off-target fecundity and mating**	22.8	-39.6	635 [214-1414]	1.00 [0.94-1.15]

Table S4E. Cages 1 and 2 combined analysis with functional (r1) resistance

Fitness cost model	Log-likelihood	AICc	Effective population size	Fitness	Relative r1 rate
**Somatic cleavage female fecundity**	53.5	-100.0	184 [104-298]	0.57 [0.47-0.69]	0 [0 - 0.0031]
**Direct viability**	50.8	-94.6	172 [97-278]	0.75 [0.66-0.85]	0 [0 - 0.0026]

Table S5 Artificial selection cage experiment

Generation	Cage 1		Cage 2		Cage 3	
	DsRed	wild-type	DsRed	wild-type	DsRed	wild-type
**0**	100	0	100	0	100	0
**1**	243	2	246	4	268	3
**2**	83	1	92	1	43	0
**3**	21	0	16	0	0	0
**4**	0	0	0	0	0	0
